# Laparoscopic resection of idiopathic jejunal arteriovenous malformation after metallic coil embolization

**DOI:** 10.1186/s40792-018-0486-4

**Published:** 2018-07-18

**Authors:** Makiko So, Yoshiro Itatani, Kazutaka Obama, Shigeru Tsunoda, Shigeo Hisamori, Kyoichi Hashimoto, Yoshiharu Sakai

**Affiliations:** Department of Surgery, Graduate School of Medicine, Kyoto University, Kyoto, 606-8507 Japan

**Keywords:** Laparoscopy, Jejunal AVM, Metallic coil embolization

## Abstract

**Background:**

Arteriovenous malformations (AVM) developed in the small intestine are rare, and it is sometimes difficult to identify and treat bleeding from small intestinal AVMs endoscopically because of their localization. We present a case of a jejunal AVM successfully treated with the combination of metallic coil embolization and laparoscopic surgery.

**Case presentation:**

A 50-year-old woman with a history of repetitive gastrointestinal bleeding was admitted to the hospital. Selective angiography revealed a jejunal AVM that was treated with metallic coil embolization. However, the lesion rebled 3 months later, and it was embolized again with metallic coils. Considering the risk of rebleeding, we performed laparoscopic resection of the jejunal AVM. Under laparoscopy alone, it was impossible to detect the lesion of the AVM. We used X-ray fluoroscopy intraoperatively to detect the metallic coils at the AVM. Partial resection of the jejunum with the AVM was performed followed by functional end-to-end anastomosis. The patient was discharged from the hospital without any complications after the surgery.

**Conclusions:**

The combination of metallic coil embolization by angiography and laparoscopic surgery with X-ray fluoroscopy can be effective for patients with repetitive bleeding from jejunal AVM.

## Background

Gastrointestinal arteriovenous malformations (AVM) often cause gastrointestinal bleeding, but sometimes they are difficult to diagnose by endoscopy in the absence of active bleeding. In particular, a small intestinal AVM cannot be diagnosed by either upper or lower endoscopy because of its location, even if it is actively bleeding. The diagnosis of small intestinal AVMs is usually made by a selective mesenteric arteriography, demonstrating characteristic vascular tufts and very early venous phase [1]. In 1986, Kandarpa et al. reported the efficacy of coil embolization under angiography just before surgery to localize intestinal AVMs [2]. Although an endoscopic examination is effective when the lesion is accessible, a selective mesenteric angiography is the standard examination to diagnose, localize, and treat a small intestinal AVM. If the bleeding is uncontrollable by either endoscopy or angiographic embolization, surgical resection of the intestine affected is necessary. We present here a rare case of a jejunal AVM successfully treated with the combination of metallic coil embolization and laparoscopic surgery, and reviewed cases of small intestinal AVM resected laparoscopically.

## Case presentation

**Fig. 1:**
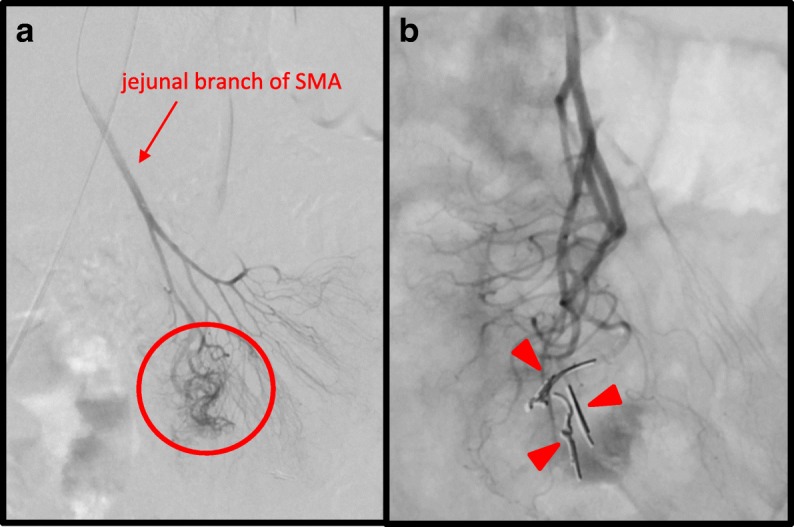
Selective angiography of the SMA. **a** Extravasation (red circle) from the jejunal branch of the SMA (red arrow) supplying the AVM is clearly observed. **b** Three vasa recta branches of the jejunal branch embolized with metallic coils (red arrowheads), with no extravasation from the AVM evident after coil embolization

**Fig. 2:**
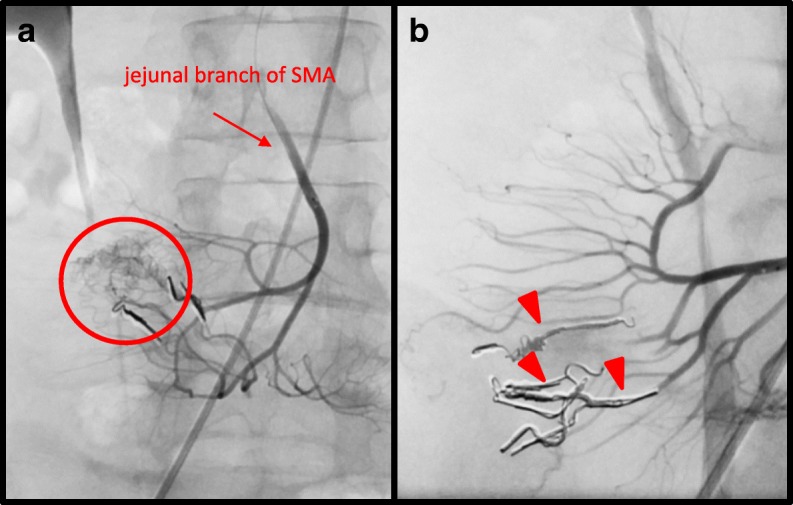
Second selective angiography of SMA (red arrow). **a** Extravasation (red circle) detected at the same AVM embolized during the first angiography. Red arrow indicates the jejunal branch of the SMA. **b** Additional metallic coils (red arrowheads) embolized to 3 vasa recta branches close to the previously embolized arteries, with no extravasation evident after embolization

A 50-year-old woman presented to our hospital with hematochezia and anemia. 1 year earlier, she experienced severe anemia (hemoglobin 4.0 g/dL) that was treated with a blood transfusion at another hospital. The diagnosis at that time was a hemorrhagic gastric ulcer, and she was treated with a proton pump inhibitor. Contrast-enhanced abdominal computed tomography (CT) done just before the first administration to our hospital showed multiple liver lesions of arterioportal and portal venous shunts, hemangiomas, and a large focal nodular hyperplasia. She had hematochezia and anemia (hemoglobin 7.0 g/dL) again and was referred to our hospital for further examination. Upper and lower gastrointestinal endoscopies including double-balloon enteroscopy did not reveal any bleeding lesions in her esophagus, stomach, duodenum, proximal jejunum, colon, or rectum, although she had grade 1 esophageal varices. Angiographic examination revealed an AVM, with signs of extravasation, at the jejunal branch of the superior mesenteric artery (SMA; Fig. 1a). Three vasa recta branches of the jejunum at the AVM lesion were embolized with metallic coils to stop the bleeding (Fig. 1b). The patient was then discharged from the hospital without any complications. Three months after the embolization, she experienced hematochezia and anemia again and was admitted to our hospital. Repeat angiography showed rebleeding from the same AVM, and an additional 3 vasa recta branches were treated with metallic coil embolization (Fig. 2). The coil embolization was temporarily successful again. However, because of the risk of another rebleeding from the same AVM in addition to the risk of necrosis of the coil-embolized jejunum, we considered resection of the affected jejunum to be the optimal treatment and recommended this to the patient.

**Fig. 3:**
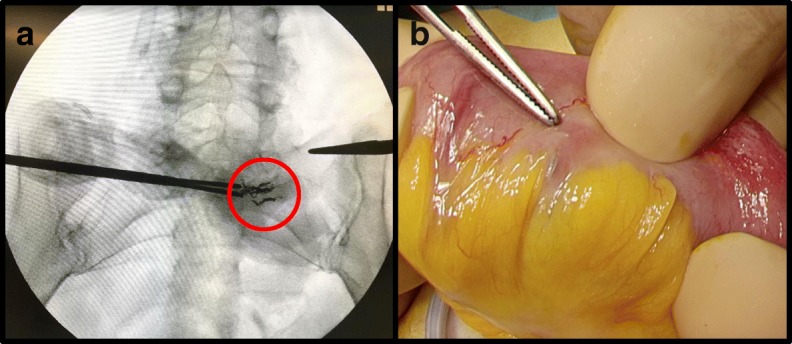
Intraoperative detection of metallic coils. **a** X-ray fluoroscopy localizing the metallic coils (red circle) embolized under the previous angiographies. **b** Forceps indicating one of the metallic coils after taking out of the lesion detected under X-ray fluoroscopy

**Fig. 4:**
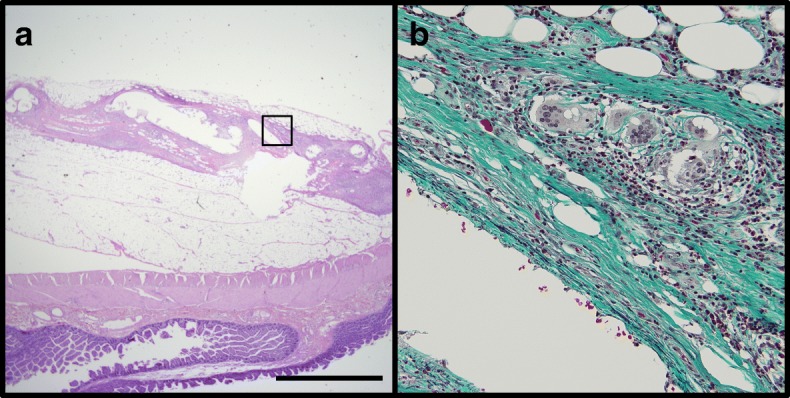
Pathological examinations of the resected tissue. **a** Hematoxylin and eosin staining reveals thickening of the vessels in the mesentery. Scale bar, 200 μm. **b** Elastica-Masson staining reveals collagenous fibrosis (green) of the vessel walls. Inflammatory cells such as lymphocytes, polymorphonuclear cells, and multinucleated giant cells are infiltrating around the vessels, suggesting focal inflammation associated with the coil embolization

Elective laparoscopic surgery was performed under general anesthesia. Although initial investigation under laparoscopy alone failed to localize the lesion, X-ray fluoroscopy showed a clear image of the metallic coils embolizing the AVM (Fig. 3a). Subsequently, the small bowel was taken out through the umbilical incision, and the metallic coils were confirmed by palpation under direct vision. Partial resection of the jejunum was performed, followed by functional end-to-end anastomosis using linear staplers (Fig. 3b). Pathological examination revealed fibrous thickening of the vessels and infiltration of inflammatory cells in the mesentery, suggesting a focal inflammation in response to the coil embolization (Fig. 4). There was no necrotic intestine caused by the embolization (Fig. 4). She had no complications after surgery and was discharged within 1 week. She did not have any hematochezia after resection of the AVM during 8 months of follow-up.

## Discussion

AVM represents an abnormal connection of arteries and veins. It occurs mostly in the head and neck, including in the central nervous system, but can appear in any location in the body. In the gastrointestinal tract, it can cause acute and chronic bleeding, which can be fatal. A gastrointestinal AVM can be diagnosed by angiography, showing abnormal vessels as a small vascular tuft, usually fed by a single artery [1]. These arteries demonstrate rapid filling, tortuous, dilated, and opaque terminal “berry”-like structures, accompanied with early filling of relatively enlarged draining veins [3]. An endoscopic approach is effective for AVMs located in the esophagus, stomach, duodenum, colon, or rectum [4]. On the other hand, small intestine AVMs are very difficult to reach by endoscopy and there are few case reports in which small intestine endoscopies were used to detect them [5, 6]. In our case, we did try a small intestine endoscopy, but failed to detect any bleeding lesions.

Once patients need surgery for the treatment of small intestine AVMs, it can be difficult to localize the lesions during surgery. To overcome this problem, several intraoperative techniques have been reported. Evans et al. reported the effectiveness of measuring intraoperative mesenteric venous pressure and PO2 [7]. In this approach, the venous return from the AVM was characterized by an elevated venous pressure and PO2 levels compared with those from adjacent normal intestines. The same group also reported the intraoperative use of Doppler ultrasound to detect intestinal AVMs [8]. Defreyne et al. reported the intraoperative use of a methylene blue dye injection to visualize the location of small intestine AVM [9]. Recently, Ono et al. reported that an intraoperative indocyanine green dye injection and examination with a fluorescent scope could localize jejunal AVMs [10]. Although these techniques described above are useful in open surgery, they are sometimes difficult to be performed and observed during laparoscopic surgery with limited view area on a screen. As mentioned above, coil embolization prior to surgery is also useful for the localization of small intestine AVMs because the coil is palpable intraoperatively in open surgery, and detectable under X-ray fluoroscopy in both open and laparoscopic surgery.

In addition to the useful technique for the localization intraoperatively, coil embolization can also be a powerful method for treating acute bleeding from the AVM. In our case, we employed this method at the patient’s first presentation at our hospital and were able to stop bleeding from the AVM without any ischemic changes to the jejunum. Unfortunately, however, the same AVM rebled 3 months later and was successfully treated with a second coil embolization. Although there seems to be no consensus about how many vasa recta branches can be embolized in case of small intestinal bleeding, it is obvious that an excess amount of embolization would result in acute or chronic ischemia of the intestine. In our case, the patient did not show any acute complications after the second embolization, although a total of six vasa recta branches were embolized.

There are 13 cases (12 reports) of small intestinal AVM resected under laparoscopic surgery (Table 1). Among them, five cases were pediatric, suggesting that they were congenital and relatively large, and the AVMs were directly visible intraoperatively [11–14]. As for other eight adult cases, they used tools described above for intraoperative detection during laparoscopic surgery in most of the cases [6, 9, 15–19]. If the AVMs were accessible by double-balloon enteroscopy, it would be a powerful technique for localization of small intestinal AVMs either by clipping or tattooing, or direct observation intraoperatively. On the other hand, if the AVMs could not be observed by endoscope, angiographic approach must be essential for localization. Although intraoperative injection of methylene blue could visualize the lesion of AVM, it needs catheterization during operation, which requires additional patient care in the operation room. On the other hand, embolized coils can be left in the branches of mesenteric artery close to AVM for both treatment and localization purpose after angiography, which gives us time to prepare the best preoperative management for the patients.

**Table 1:** Previous reports of small intestinal AVMs resected laparoscopically

Authors	Title	Intraoperative detection of AVM	Article	Year
Chung CS et al. [15]	Emergent single-balloon enteroscopy for overt bleeding of small intestinal vascular malformation	Tattooing	World J Gastroenterol	2018
Kim SH et al. [11]	Vascular malformations of the small intestine manifesting as chronic anemia: two pediatric cases managed by single-site umbilical laparoscopic surgery	Visible	Int J Surg Case Rep	2017
Lee YH et al. [12]	A long-segmental vascular malformation in the small bowel presenting with gastrointestinal bleeding in a preschool-aged child	Visible	Int J Radiol	2016
Sahn B et al. [13]	Vascular malformation as a cause of occult gastrointestinal bleeding	Visible	J Pediatr Gastroenterol Nutr	2015
Fujii T et al. [5]	Arteriovenous malformation detected by small bowel endoscopy	Not described	Case Rep Gastroenterol	2014
Kalmar P et al. [14]	Large, segmental, circular vascular malformation of the small intestine (in a female toddler with hematochezia): unusual presentation in a child	Visible	BMC Pediatr	2014
Martinez JC et al. [16]	Single incision laparoscopic surgery approach for obscure small intestine bleeding localized by CT guided percutaneous injection of methylene blue	Methylene blue	Int J Surg Case Rep	2014
Fujikawa T et al. [17]	Successful resection of complicated bleeding arteriovenous malformation of the jejunum in patients starting dual-antiplatelet therapy just after implanting a drug-eluting coronary stent	Clip	BMJ Case Rep	2012
Mukai M et al. [18]	Intraoperative fluoroscopic detection of an occult jejunal arteriovenous malformation	Methylene blue and coil	J Laparoendosc Adv Surg Tech	2006
Otsuka S et al. [6]	Resection of arteriovenous malformation of the jejunum treated by laparoscopy-assisted surgery	Intestinal endoscopy	J Jpn Surg Assoc	2002
Yamamoto T et al. [19]	Resection of the arteriovenous malformation of the jejunum with the use of laparoscopy-assisted surgery combined with marking coil	Coil	Jpn J Gastroenterol Surg	1999
Defreyne L et al. [9]	Jejunal arteriovenous malformation, diagnosed by angiography and treated by embolization and catheter-guided surgery: case report and review of literature	Methylene blue	Abdom Imaging	1998

## Conclusions

We report a rare case of jejunal AVM treated with metallic coil embolization followed by laparoscopic resection. This case suggests the effectiveness of the combination of metallic coil embolization and laparoscopic surgery for patients with repetitive bleeding from a jejunal AVM.

## Abbreviations

AVM: Arteriovenous malformation
CT: Computed tomography
SMA: Superior mesenteric artery
